# Recovery from chronic fatigue syndrome after treatments given in the PACE trial

**DOI:** 10.1017/S0033291713000020

**Published:** 2013-01-31

**Authors:** P. D. White, K. Goldsmith, A. L. Johnson, T. Chalder, M. Sharpe

**Affiliations:** 1 Wolfson Institute of Preventive Medicine, Barts and the London School of Medicine and Dentistry, Queen Mary University of London, UK; 2 Biostatistics Department, Institute of Psychiatry, King's College London, UK; 3 MRC Biostatistics Unit, Institute of Public Health, University of Cambridge, UK; 4 MRC Clinical Trials Unit, London, UK; 5 Academic Department of Psychological Medicine, King's College London, UK; 6 Department of Psychiatry, University of Oxford, UK

**Keywords:** Chronic fatigue syndrome, cognitive behaviour therapy, graded exercise therapy, randomized control trial, recovery

## Abstract

**Background:**

A multi-centre, four-arm trial (the PACE trial) found that rehabilitative cognitive behaviour therapy (CBT) and graded exercise therapy (GET) were more effective treatments for chronic fatigue syndrome (CFS) than specialist medical care (SMC) alone, when each was added to SMC, and more effective than adaptive pacing therapy (APT) when added to SMC. In this study we compared how many participants recovered after each treatment.

**Method:**

We defined recovery operationally using multiple criteria, and compared the proportions of participants meeting each individual criterion along with two composite criteria, defined as (*a*) recovery in the context of the trial and (*b*) clinical recovery from the current episode of the illness, however defined, 52 weeks after randomization. We used logistic regression modelling to compare treatments.

**Results:**

The percentages (number/total) meeting trial criteria for recovery were 22% (32/143) after CBT, 22% (32/143) after GET, 8% (12/149) after APT and 7% (11/150) after SMC. Similar proportions met criteria for clinical recovery. The odds ratio (OR) for trial recovery after CBT was 3.36 [95% confidence interval (CI) 1.64–6.88] and for GET 3.38 (95% CI 1.65–6.93), when compared to APT, and after CBT 3.69 (95% CI 1.77–7.69) and GET 3.71 (95% CI 1.78–7.74), when compared to SMC (*p* values ⩽0.001 for all comparisons). There was no significant difference between APT and SMC. Similar proportions recovered in trial subgroups meeting different definitions of the illness.

**Conclusions:**

This study confirms that recovery from CFS is possible, and that CBT and GET are the therapies most likely to lead to recovery.

## Introduction

Chronic fatigue syndrome (CFS) is a disabling disorder of unknown cause, with a prevalence of between 0.4% and 2.5% in the UK population (Prins *et al.* 2006). Myalgic encephalomyelitis (ME) is thought by some researchers to be the same disorder and by others as different with separate diagnostic criteria (Prins *et al.* 2006; NICE, 2007). Common symptoms of CFS include fatigue, painful muscles and joints, poor concentration and sleep disturbance; these symptoms do not remit with rest and are made worse by activity.

Established treatments for CFS include the rehabilitative therapies of cognitive behaviour therapy (CBT) and graded exercise therapy (GET) (NICE, 2007). Several meta-analyses of these therapies indicate moderate benefit from these treatments (Edmonds *et al.* 2004; Malouff *et al.* 2008; Price *et al.* 2008; Castell *et al.* 2011). The recently published PACE trial found that CBT and GET were more effective in reducing both fatigue and physical disability than adaptive pacing therapy (APT), when each was added to specialist medical care (SMC), and more effective than SMC alone (White *et al.* 2011).

Although the PACE trial found that many patients improved with CBT and GET, the question of how many patients recovered remains unanswered. We know that recovery from CFS without treatment is reported to be uncommon; a systematic review found that a median (range) of only 7% (0–48%) recovered over time (Cairns & Hotopf, 2005). We also know from a previous study that 24% of 25 patients rated themselves as ‘recovered’ 5 years after CBT (Deale *et al.* 2001) and that when applying more detailed, operationalized criteria (no longer fatigued, able to resume activities, and a perception of health and fatigue similar to that of a healthy person), 23% of 96 patients were rated as ‘recovered’ immediately after a course of CBT (Knoop *et al.* 2007). However, there have been no published reports comparing the proportions recovered after CBT with those achieved after other treatments.

Before we can determine the proportions recovered we need an operational definition of recovery itself. An ideal definition remains uncertain, as is the case for other conditions, such as low back pain (Kamper *et al.* 2011). Measurement of recovery could involve many domains. Within the trial context these could include: no longer meeting trial eligibility criteria, not having significant symptoms, not being disabled by the illness, and regarding one's health as having improved considerably. Within a clinical context, the additional criteria of not meeting alternative definitions of CFS and ME could also be applied (Sharpe *et al.* 1991; Tyrrell, 1994; Reeves *et al.* 2003).

Creating criteria for recovery from domains that are measured on a continuum requires the setting of operational thresholds based on population studies or trial eligibility criteria (Powell *et al.* 2004; Knoop *et al.* 2007; Malouff *et al.* 2008). In this context it is important to note that recovery does not mean being free of all symptoms; population studies show that the average person in the UK reports a mean of four symptoms in any 2-week period (McAteer *et al.* 2011). The three most common symptoms reported were fatigue, headache and joint pain; symptoms consistent with CFS (McAteer *et al.* 2011). Recovery may be taken to imply that the patient has made a transition from ill health to remission and also is at little risk of recurrence (Nisenbaum *et al.* 2003). Although we can measure remission, we cannot be certain of the risk of recurrence without long-term follow-up; we therefore use the term ‘recovery’ in this paper to mean recovery from the current episode of the illness.

The aims of this study were to: (*a*) define operationalized criteria for recovery on relevant domains, (*b*) calculate the proportions of trial participants meeting each of these individual criteria in each treatment arm, (*c*) calculate the proportion of trial participants meeting all the recovery criteria to provide a comprehensive and conservative definition of recovery in each treatment arm, (*d*) compare the proportions meeting both trial and clinical recovery criteria between the treatment arms, and (*e*) compare these proportions within each of the two subgroups of participants in the trial, which met the international definition of CFS and the London definition of ME (Tyrrell, 1994). As CBT and GET were the most effective treatments in the trial, we hypothesized that they would also be associated with greater proportions of recovered individuals at the 52-week primary end-point than either APT or SMC alone.

## Method

The PACE trial recruited 641 participants from six secondary care CFS clinics in England and Scotland, allocated randomly to one of four treatment groups, with a final follow-up 52 weeks after randomization (White *et al.* 2007, 2011). All participants met the Oxford criteria for CFS (Sharpe *et al.* 1991). The four trial treatment arms were: SMC alone delivered by specialist CFS doctors, SMC plus APT delivered by occupational therapists, SMC plus CBT delivered by clinical psychologists, and SMC plus GET delivered by physiotherapists. Specialist doctors gave an explanation of why participants were ill and general advice about managing the illness. They also prescribed medicines to help with symptoms such as insomnia and pain, or advised general practitioners (GPs) on which medicine was appropriate. If a participant was randomized to this treatment alone, they were also encouraged to use self-help management that made most sense to them. APT involved carefully matching activity levels to the amount of energy available. Therapists worked with participants in this group to help monitor activity and symptoms, aiming to improve quality of life and create the best conditions for natural remission. CBT involved examining how thoughts, behaviour and symptoms interact with each other. Between therapy sessions, participants in this group were encouraged to try out new ways of coping with their illness. GET involved gradually increasing physical activity to improve fitness and get the body used to activity again. Therapists helped participants in this group to work out a basic activity routine and slowly build up the amount of exercise (White *et al.* 2007, 2011).

### Domains, measures and criteria for defining recovery

We chose domains for defining recovery on the basis of the previous literature and the measures available from the trial. The thresholds defining our criteria for recovery on each domain were based either on population normal ranges, case definitions or trial entry criteria. We changed three of the thresholds for measuring recovery from our original protocol (White *et al.* 2007) before the analysis, as explained below.

#### Fatigue: the Chalder Fatigue Questionnaire (CFQ)

The 11-item CFQ measured the severity of symptomatic fatigue, rated by the participant, and was one of the primary outcomes of the trial (Chalder *et al.* 1993). The respondent chose from one of four answers (‘less than usual’, ‘no more than usual’, ‘more than usual’ and ‘much more than usual’) to each item, scores being 0, 1, 2 or 3, with a maximum score of 33 indicating severe fatigue. We changed our original protocol's threshold score for being within a normal range from a binary score of ⩽3 out of 11 (White *et al.* 2007), which represented a screening threshold for abnormal fatigue from a small primary care study (Chalder *et al.* 1993), following the publication of a much larger study of fatigue in adults in a representative population sample of patients registered with a GP from South East England (Cella & Chalder, 2010). This showed a population mean (s.d.) Likert score of 14.2 (4.6) out of a maximum score of 33. We therefore considered a score of 18 (highest integral score below the mean plus 1 s.d.) or less as within the normal range for fatigue.

#### Physical function: the 36-Item Short-Form Health Survey (SF-36) physical function subscale

The SF-36 physical function subscale, rated by the participants, was the other primary outcome from the trial (McHorney *et al.* 1993). The scale asks about 10 aspects of physical function, such as the ability to walk 100 m, with three possible answers: not limited, limited a little, and limited a lot. This provides a derived score that ranges from worst (0) to best possible function (100). We changed our original protocol's threshold score for being within a normal range on this measure from a score of ⩾85 to a lower score as that threshold would mean that approximately half the general working age population would fall outside the normal range. The mean (s.d.) scores for a demographically representative English adult population were 86.3 (22.5) for males and 81.8 (25.7) for females (Bowling *et al.* 1999). We derived a mean (s.d.) score of 84 (24) for the whole sample, giving a normal range of 60 or above for physical function.

#### CFS case definition: Oxford criteria

This was the definition of CFS used to define eligibility for participation in the trial. Research assessors judged whether participants still met Oxford criteria for CFS at 52 weeks; specifically they determined if: (1) fatigue was the main symptom, (2) it was of definite onset and not lifelong, (3) fatigue was severe, disabling and affected physical and mental function, and (4) fatigue had persisted for 6 months or more and was present 50% of the time (Sharpe *et al.* 1991). To satisfy the third criterion for severity of fatigue and disability, participants had to meet trial entry thresholds for fatigue (a binary score of ⩾6 out of 11 on the CFQ) and abnormal levels of physical function (a score of ⩽65 out of 100 on the SF-36 physical function subscale) (White *et al.* 2007).

#### CFS case definition: the International (Centers for Disease Control and Prevention, CDC) criteria

The research assessor used participant ratings to judge whether participants met the International (CDC) criteria for CFS at 52 weeks (Reeves *et al.* 2003), which included: (1) severe chronic fatigue for at least 6 months with other known medical conditions (whose manifestation includes fatigue) excluded by clinical diagnosis; and (2) concurrently have four or more of the following symptoms: post-exertional malaise, impaired memory or concentration, unrefreshing sleep, muscle pain, multi-joint pain without redness or swelling, tender cervical or axillary lymph nodes, sore throat, headache. For the purposes of this study, the four or more symptoms needed to be present within the previous week of the assessment date, rather than the previous 6 months (Reeves *et al.* 2003). To meet the first criterion for severity, participants had to have abnormal levels of fatigue, which we took to be the trial entry eligibility criteria for the CFQ, and abnormal levels of physical function (as above) (White *et al.* 2007).

#### ME case definition: the London criteria

Research assessors judged whether participants met the London criteria for ME at 52 weeks (Tyrrell, 1994). Specifically, these criteria included: (1) exercise-induced fatigue precipitated by trivially small exertion, (2) impairment of short-term memory and loss of powers of concentration, (3) fluctuations of symptoms usually precipitated by physical or mental exertion, (4) symptoms present for at least 6 months, and (5) no ‘primary’ depressive illness and no anxiety disorder present (which we interpreted as no co-morbid mood disorder of any kind). To standardize thresholds for severity with other case definitions, participants also had to meet trial entry eligibility criteria for the CFQ and abnormal levels of physical function (as above) (White *et al.* 2007).

#### Overall change in health: the Clinical Global Impression (CGI) change score

The self-rated CGI change score (range 1–7) provided a participant-rated global measure of overall health change, not just change in CFS (Guy, 1976). We considered scores of 1 (‘very much better’) or 2 (‘much better’) as evidence of the process of recovery, rather than our original protocol threshold of a score of 1 only, because we considered that participants rating their overall health as ‘much better’ represented the process of recovery. The CGI change scale was also rated by the SMC doctor at the 52-week review. These scores were used as imputed scores when the participant-rated CGI score was missing at 52 weeks (*n* = 22).

#### Composite definitions of recovery

We operationalized two composite definitions of recovery: (1) trial recovery from CFS, and (2) clinical recovery from the illness, however it was defined. To provide a definition of trial recovery, we calculated a hierarchical, cumulative definition that included the following domains mentioned earlier: normal range in fatigue, normal range in physical function, not meeting the Oxford case definition of CFS, and CGI scores of 1 or 2 (‘very much’ or ‘much’ better). To fulfil the criteria for clinical recovery from the illness, participants had to meet all the criteria for trial-defined recovery (described earlier), in addition to not meeting either the International (CDC) criteria for CFS or the London criteria for ME.

### Analysis

We reported descriptive statistics (percentage and frequency) in each treatment arm for each individual domain of recovery. We then gave the results of a cumulative hierarchy of the proportions meeting domains of trial recovery in the order of: normal ranges for both the fatigue and physical function scores, not meeting the Oxford criteria for CFS, and the clinical global impression positive change scores (1 or 2). The cumulative hierarchy of clinical recovery was then applied as the trial definition of recovery combined with not meeting criteria for either the International (CDC) definition of CFS or the London criteria for ME. We calculated the number needed to treat (NNT) for one extra participant to recover by dividing 100 by the proportion recovering after either CBT or GET minus the proportion recovering after SMC, rounded up to the nearest whole number.

To examine recovery in participants who also met either the International (CDC) definition of CFS or the London definition of ME at entry to the trial, we applied the same cumulative hierarchy of criteria in these subgroups. We then used logistic regression to compare the odds of recovery between trial arms, using the originally hypothesized comparison groups: APT *v*. SMC, CBT *v*. SMC, GET *v*. SMC, APT *v*. CBT and APT *v.* GET (White *et al.* 2011). Resulting odds ratios (ORs) and 95% confidence intervals (CIs) were adjusted for the stratification variables of centre, meeting International (CDC) criteria at baseline, meeting the London definition of ME at baseline, and having a depressive illness at baseline. Interaction terms for trial arm by meeting either CFS or ME criteria at baseline were used to calculate the odds of recovery in (1) the subgroup meeting International (CDC) criteria at baseline and (2) the subgroup meeting the London definition of ME at baseline.

## Results

We studied 640 participants (excluding one participant who withdrew consent after the study). The mean (s.d.) age was 38 (12) years, 77% were female, and 93% were Caucasian. All participants met the Oxford criteria for CFS; 67% of participants also met the International (CDC) criteria for CFS and 51% met the London criteria for ME (White *et al.* 2011). The median (interquartile range, IQR) duration of illness was 32 (16–68) months, 47% had a co-morbid psychiatric condition at randomization (33% depressive disorder). By 52 weeks, only 33 (5%) were missing primary outcome data, with no significant difference between treatment groups.

Table 1 *a* shows the individual criteria for recovery at 52 weeks. Whatever the domain, the largest proportions of participants recovering had received either CBT or GET. Overall, the largest proportions of participants meeting criteria for recovery were those who no longer met criteria for ME, followed by the Internationally defined criteria for CFS, and then the Oxford-defined criteria for CFS.

**Table 1.** Participants, % (n/total), meeting criteria for recovery

Domains and measures	APT (159)	CBT (161)	GET (160)	SMC (160)
(a) Individual criteria				
Fatigue				
Within CFQ normal range	22 (34/153)	41 (60/148)	33 (51/154)	21 (32/152)
Physical function				
Within SF-36-PF normal range	35 (53/153)	52 (77/148)	53 (81/154)	41 (62/152)
Both fatigue and function				
Within both CFQ and SF-36-PF normal ranges	16 (25/153)	30 (44/148)	28 (43/154)	15 (22/152)
Case criteria				
CDC criteria not met	49 (74/150)	67 (97/144)	65 (93/144)	51 (76/149)
Oxford criteria not met	43 (64/149)	54 (77/143)	56 (81/144)	41 (62/150)
London ME criteria not met	68 (100/147)	76 (107/140)	77 (106/138)	66 (97/148)
Overall health change				
CGI 1 or 2	30 (48/158)	40 (62/154)	40 (63/156)	25 (40/158)
	APT	CBT	GET	SMC
(b) Composite criteria for both trial and clinical recovery (combined hierarchically)				
Cumulative criteria for trial recovery				
Both CFQ and SF-36-PF in normal range	16 (25/153)	30 (44/148)	28 (43/154)	15 (22/152)
And Oxford criteria not met	15 (23/149)	28 (40/143)	28 (41/144)	14 (21/150)
And CGI 1 or 2 (95% CI)	8 (12/149) (4–14)	22 (32/143) (16–30)	22 (32/143) (16–30)	7 (11/150) (4–13)
Additional criteria for clinical recovery				
And International (CDC) CFS criteria not met	8 (12/149)	22 (32/143)	22 (32/143)	7 (11/149)
And London ME criteria not met (95% CI)	8 (12/147) (4–14)	21 (29/139) (14–29)	21 (29/138) (15–29)	7 (11/147) (4–13)

CFQ, Chalder Fatigue Questionnaire; SF-36-PF, Short Form 36-item measure of physical function; CGI, Clinical Global Impression change measure; APT, adaptive pacing therapy; CBT, cognitive behaviour therapy; GET, graded exercise therapy; SMC, specialist medical care; CDC, Centers for Disease Control and Prevention; CFS, chronic fatigue syndrome; ME, myalgic encephalomyelitis; CI, confidence interval.

Normal range for CFQ was ⩽18/33; normal range for SF-36-PF was ⩾60/100.

Values given as % (*n*/total).

Table 1*b* shows the hierarchical, cumulative definitions for both trial and clinical recovery. As each additional criterion was added, the proportions meeting criteria for recovery generally were reduced. For all the criteria applied, the largest proportions of recovered participants were found in those who had received either CBT or GET. Some data were missing for 6% of those receiving APT and SMC and 11% for those in receipt of CBT and GET. The NNT for one extra participant to achieve trial recovery was 7 for both CBT and GET.

The proportions meeting criteria for clinical recovery from the illness were very similar to the proportions meeting the trial definition for recovery (Table 1*b*). Although it seemed that slightly smaller proportions had recovered from the illness as a whole, when the criterion ‘not meeting the London criteria for ME’ was applied, we found that the differences were due to missing data rather than to change in recovery status. For this reason, we made a *post-hoc* decision to model the more complete data set of those meeting the trial definition of recovery rather than the illness definition of recovery.

Table 2 shows the proportions who met the trial definition of recovery in subgroups that met alternative definitions of CFS or ME at baseline. The pattern of results was very similar to those for all participants; CBT and GET were associated with the largest proportions of participants recovered.

**Table 2.** Composite criteria for trial recovery in subgroups meeting alternative definitions of CFS or ME at baseline

	APT	CBT	GET	SMC
All participants	8 (12/149)	22 (32/143)	22 (32/143)	7 (11/150)
International (CDC) criteria	9 (9/102)	19 (17/89)	22 (20/93)	6 (6/98)
London ME criteria	11 (8/75)	21 (15/70)	21 (16/75)	10 (7/73)

CFS, Chronic fatigue syndrome; ME, myalgic encephalomyelitis; APT, adaptive pacing therapy; CBT, cognitive behaviour therapy; GET, graded exercise therapy; SMC, specialist medical care; CDC, Centers for Disease Control and Prevention.

Values given as % (*n*/total).

Table 3 shows that odds of trial definitions of recovery after either CBT or GET were more than three times those after either APT or SMC. There was no significant difference between APT and SMC. A similar pattern of differences was observed in the two subgroups that met the International (CDC) definition for CFS (interaction term *p* value = 0.77) and in those who met the London criteria for ME at entry (interaction term *p* value = 0.76).

**Table 3.** Comparison of odds for composite trial recovery adjusted for baseline characteristics

	All participants		Met international (CDC) criteria at baseline		Met London ME criteria at baseline	
	OR (95% CI)	*p* value	OR (95% CI)	*p* value	OR (95% CI)	*p* value
CBT *v.* APT	3.36 (1.64–6.88)	0.001	2.73 (1.16–6.44)	0.022	2.72 (1.09–6.78)	0.032
CBT *v.* SMC	3.69 (1.77–7.69)	<0.001	4.14 (1.56–11.00)	0.004	3.18 (1.23–8.23)	0.017
GET *v.* APT	3.38 (1.65–6.93)	0.001	2.96 (1.27–6.90)	0.012	2.52 (1.01–6.28)	0.048
GET *v.* SMC	3.71 (1.78–7.74)	<0.001	4.50 (1.72–11.79)	0.002	2.95 (1.14–7.61)	0.026
APT *v.* SMC	1.10 (0.47–2.58)	0.83	1.52 (0.52–4.46)	0.450	1.17 (0.40–3.43)	0.77

CBT, Cognitive behaviour therapy; APT, adaptive pacing therapy; SMC, specialist medical care; GET, graded exercise therapy; CDC, Centers for Disease Control and Prevention; ME, myalgic encephalomyelitis; OR, odds ratio; CI, confidence interval.

International (CDC) interaction *p* = 0.77; London ME interaction *p* = 0.76.

## Discussion

We found that CBT and GET were both significantly more likely than APT and SMC to be associated with recovery at 52 weeks, even when using a conservative definition of recovery. Between a fifth and a quarter of participants were recovered by 52 weeks after either CBT or GET, with an NNT of seven. A similar pattern was seen in the two subgroups meeting alternative definitions for CFS and ME at entry into the trial.

The main limitation of this analysis is the absence of a generally agreed measure of recovery. We addressed this by using multiple domains of health and disability. The domains chosen and the criteria for recovery on each were defined before we undertook the analysis. Alternative domains could have been used, such as return to work or objective measures of physical activity. Return to work is not, however, an appropriate measure of recovery if the participant was not working before their illness and is influenced by other factors such as the job market. Objective measures of physical activity have been found previously to correlate poorly with self-reported outcomes (Wiborg *et al.* 2010), which may be related to the finding that activity patterns in CFS patients are heterogeneous, with only a minority being pervasively passive (van der Werf *et al.* 2001). We did not include any measures of mood in our domains of recovery as mood is not part of the definition of the illness.

The amount of missing outcome data was greater after CBT and GET than after APT and SMC, but the percentages missing were small enough not to warrant sensitivity analyses, particularly because all but 33 (5%) participants contributed some data. The prevalence of the case-level International (CDC) definition of CFS may have been inaccurate because we only examined for accompanying symptoms in the previous week, not the previous 6 months. The assessments of caseness (CDC, London and Oxford criteria) relied on a mixture of self-ratings and research assistant assessments, making some observer bias possible. We changed some of the thresholds for measuring recovery from those of the original protocols (White *et al.* 2007); we made the changes before analysis and to more accurately reflect recovery. Our finding that only 7% recovered after the minimal treatment of SMC, exactly the same proportion as the median recovery rate found without treatment (Cairns & Hotopf, 2005), supports these revised thresholds. Finally, we cannot be sure that recovery was sustained beyond the assessment at 52 weeks.

How do these results compare with previous studies? We are not aware of any previous studies that have compared comprehensively defined recovery between different treatments. Two studies of recovery in adults after CBT found similar proportions in recovery: 23% and 24% (Deale *et al.* 2001; Knoop *et al.* 2007), compared with 22% in the PACE trial. One of these studies had a 5-year follow-up period rather than the 1 year of our study, and the majority had received further treatment in those extra 4 years, all patients being treated at one specialist CFS centre (Deale *et al.* 2001). The other study used similar criteria and domains for recovery (Knoop *et al.* 2007), but the definition for normal range used was the more liberal population mean ±2 s.d. rather than the more conservative 1 s.d. that we used; the treatment was delivered by therapists in one specialist CFS centre and outside of a trial setting. A meta-analysis of randomized controlled trials of CBT for CFS reported that a mean of 50% of the patients improved to the point of no longer being clinically fatigued (Malouff *et al.* 2008). A 2-year follow-up study after an educational intervention to encourage GET found that 55% of the treated patients no longer fulfilled trial criteria for CFS (Powell *et al.* 2004). Although not directly comparable, we found that 41% and 33% were within the population range for fatigue after CBT and GET respectively, although these proportions drop further when added to functional improvement; 54% and 56% of participants no longer met the trial entry (Oxford) case definition for CFS after CBT and GET. Our finding that 22–56% of participants met various composite or single criteria for recovery or improvement a year after starting either CBT or GET is therefore consistent with previously published studies. The NNT of 7 for recovery after both CBT and GET is within the range of the effects found for drug treatments in both general medical and psychiatric conditions (Leucht *et al.* 2012). Although only 22% recovered after either CBT or GET, if different participants recovered after CBT than after GET, then the proportion recovering after either treatment would be larger than 22%, but not larger than 39%. Recovery after CBT may be better in adolescents (Nijhof *et al.* 2012). The 7% and 8% recovered after both APT and SMC were similar to the 7% reported in a systematic review after no treatment, suggesting a lack of efficacy of these treatments (Cairns & Hotopf, 2005).

The proportions recovered in each treatment arm were similar in the subgroups meeting alternative definitions of CFS and ME, implying that these findings generalize to different definitions of CFS and ME. Patients who have either CFS or ME characterized by a principal complaint of fatigue, and who are attending out-patient clinics, should therefore be offered either CBT or GET to provide the best chance of recovery with these treatments.

As a little more than a fifth of participants treated with CBT or GET had recovered a year after starting treatment, we still need to consider ways of enhancing the effectiveness of these treatments. Two ways of doing this could be to increase the number of sessions above that offered in the PACE trial (15 sessions), because a recent meta-analysis found that higher numbers of sessions improved efficacy (Castell *et al.* 2011), or enhancing delivery of therapy, such as over the internet (Nijhof *et al.* 2012). Another approach may be to offer both CBT and GET in series. A different approach would be to identify the factors that mediate the effect of these treatments, with the aim of optimizing their effectiveness; the mediation analysis of the PACE data is under way. CFS is a heterogeneous condition and we need to find ways of identifying subgroups that respond best to each type of therapy (Cella *et al.* 2011). Finally, we also need to develop additional forms of treatment.

In conclusion, recovery from CFS is more likely to occur when CBT or GET is added to SMC than after adding APT or giving SMC by itself. The relatively small proportion of recovered patients may reflect the heterogeneity of CFS; it should also spur us on both to enhance currently available therapies and to develop new and better treatments.

## Appendix The PACE Trial Management Group

The PACE Trial Management Group consisted of the authors of this paper plus (in alphabetical order): B. J. Angus, H. Baber, J. Bavinton, M. Burgess, L. V. Clark, D. L. Cox, J. C. DeCesare, E. Feldman, P. McCrone, G. Murphy, M. Murphy, H. O'Dowd, T. Peto, L. Potts, R. Walwyn, D. Wilks.
